# Association between service scope of primary care facilities and patient outcomes: a retrospective study in rural Guizhou, China

**DOI:** 10.1186/s12913-021-06877-4

**Published:** 2021-08-28

**Authors:** Zhong Li, Meng Shi, Ruibo He, Mei Zhang, Chi Zhang, Xinyu Xiong, Liang Zhang, Boyang Li

**Affiliations:** 1 School of Medicine and Health Management, Tongji Medical College, Huazhong University of Science and Technology, Wuhan, Hubei China; 2 School of Finance and Public Administration, Hubei University of Economics, Wuhan, Hubei China

**Keywords:** Service scope, primary care facilities, utilization, expenditure, quality of care, rural China

## Abstract

**Background:**

Extending service scope of primary care facilities (PCFs) has been widely concerned in China. However, no current data about association between service scope of PCFs with patient outcomes are available. This study aims to investigate association between service scope of PCFs and patient outcomes.

**Methods:**

A multistage, stratified clustered sampling method was used to collect information about service scope of PCFs from rural Guizhou, China. Claim data of 299,633 inpatient cases covered by 64 PCFs were derived from local information system of New Rural Cooperation Medical Scheme. Service scope of PCFs was collected with self-administrated questionnaires. Primary outcomes were (1) level of inpatient institutions, (2) length of stay, (3) per capita total health cost, (4) per capita out-of-pocket cost, (5) reimbursement ratio, (6) 30-day readmission. A total of 64 PCFs were categorized into five groups per facility-level service scope scores. Generalized linear regression models, logistic regression model, and ordinal regression model were conducted to identify association between service scope of PCFs and patient outcomes.

**Results:**

On average, the median service scope score of PCFs was 20, with wide variation across PCFs. After controlling for demographic and clinical characteristics, patients living in communities with PCFs of greatest service scope (Quintile V vs. I) tended to have smaller rates of admission by county-level hospitals (-6.2 % [-6.5 %, -5.9 %], city-level hospitals (-1.9 % [-2.0 %, -1.8 %]), and provincial hospitals (-2.1 % [-2.2 %, -2.0 %]), smaller rate of 30-day readmission (-0.5 % [-0.7 %, -0.2 %]), less total health cost (-201.8 [-257.9, -145.8]) and out-of-pocket cost (-210.2 [-237.2, -183.2]), and greater reimbursement ratio (2.3 % [1.9 %, 2.8 %]) than their counterparts from communities with PCFs of least service scope.

**Conclusions:**

Service scope of PCFs varied a lot in rural Guizhou, China. Greater service scope was associated with a reduction in secondary and tertiary hospital admission, reduced total cost and out-of-pocket cost, and 30-day readmission and increased reimbursement ratio. These results raised concerns about access to care for patients discharged from hospitals, which suggests potential opportunities for cost savings and improvement of quality of care. However, further evidence is warranted to investigate whether extending service scope of PCFs is cost-effective and sustainable.

**Supplementary Information:**

The online version contains supplementary material available at 10.1186/s12913-021-06877-4.

## Background

Worldwide, to meet health needs of the ageing population and increasing non-chronic disease burden, strengthening the capacity of primary care systems by extending service scope of primary care facilities (PCFs) or hospitals has been widely concerned [1–3]. One previous study pointed out that reform on the healthcare delivery system might be a more productive solution to promote healthcare services’ appropriate use and cost-savings [4]. Historical studies have revealed that comprehensiveness of care provided by primary care physicians was associated with reduced medical expenditures, hospitalizations and emergency department visits [5], so does service scope of family physicians [6]. Meanwhile, access to after-hour services, patient-centered medical home services, urgent care centers and walk-in care could also lead to less utilization of inpatient services and unnecessary emergency department visits [7, 8]. Loosening restrictions on practice of service scope by registered nurse could also promote more significant cost savings than retail clinics did [9]. Conversely, the closure of hospital-based obstetric services in rural counties among the United States (US) has increased rates of out-of-hospital and preterm births, and births in hospitals without obstetric units [10]. Also, the service scope of primary care physicians varied a lot by health insurance schemes [11].

In rural China, healthcare systems consisted of a three-tiered system of health providers, including village clinics, township hospital, and county-level hospital [12]. Generally, there is at least one public village clinics at one village responsible for preventive and public health services, and some of basic medical care service. One public township hospital is responsible from one each township, providing preventive and public health services, and basic medical services together with village clinics, guided by the county-level hospitals. Chinese residents can freely go to health institutions of any level [12]. Patients will have a reimbursement bonus if they were referred to hospitals from PCFs [13]. One previous study in China also showed that patients from rural PCFs reported better primary care experience both in the first contact, accessibility, ongoing care, and community orientation [14]. *Law of the People’s Republic of China on Basic Healthcare and Health Promotion* requires different levels of health facilities to collaborate on the provision of preventive and public health services, clinical treatment, nursing, rehabilitation, hospice services [15]. Previous studies have focused on the determinants of service scope of PCFs. Ineffective incentives, insufficient reimbursement by the health insurance schemes have caused the closure of surgical care and obstetric care in rural facilities [13, 16]. Disproportionate proportion of direct government subsides to financial revenue is also associated with narrowed service scope [17].

Narrowed service scope of PCFs in China has been well documented [13, 16]. However, these studies have concentrated on the outcome evaluation of implementing different payment methods on the service quality, utilization of inpatient services by different level hospitals, length of stay, cost, and other patient outcomes [18–20]. Studies from the perspective of healthcare service scope remain scarce. Historic reforms and substantial investments have been tried to establish a more effective financial and administrative incentive system to promote provision and utilization of primary care services, but service scope of primary care services in China is disproportionally distributed. Also, greater proportion of public health service providers experienced burnout than clinical care providers did [21]. Preventive and public health services have been widely implemented among the PCFs [22]. However, scope of medical care services geographically varied a lot [17, 23], especially community-based mental health services, hospice care services [17, 24] and inpatient medical services [25]. For example, only a few charity hospitals and PCFs in China provided hospice care services [24]. In Shanghai, PCFs in the most urbanized areas (62.9 %) reported highest occupancy rates of hospice care beds than those PCFs in less (46.5 %) and least (30.7 %) urbanized areas [26]. One study showed that over half of mental health resources was concentrated in the Eastern China, and a few provinces like Qinghai, did not have any specialty mental health services for children or older people [27]. Those facts cause health care system fragmented and uncoordinated [2, 28, 29], which would mean unnecessary hospitalization and other undesirable treatments provided by urban hospitals and underutilized primary care services regarding curative, rehabilitative and hospice care [24, 30].

Facing these pressures, favorable policies, such as home care services to meet long-term care needs, have been introduced to strengthen the primary health care system [31], thus building a patient-centric integrated healthcare system [2]. Meanwhile, loosening restrictions on the nurse practitioners’ scope-of-practices has been piloted to meet the shortages of primary care physicians [32]. Expanding the role for the primary care system and aligning the incentives was also advocated to achieve cost-effective, high-quality care [2, 31]. However, international experiences showed that these policies would often be criticized for their potential to place inappropriate restrictions and expectations on healthcare providers [1]. Moreover, to which extend it will reduce unnecessary inpatient hospital utilization by high-level hospitals, and promote cost-savings have not been verified empirically, which are significant to improve the cost-effectiveness of intervention programs for strengthening the primary care delivery system. Therefore, this study aims to investigate association between service scope of PCFs and utilization of inpatient services, quality of care and its cost.

## Method

### Study design and data collection

Guizhou province, with a 176.1 thousand square kilometers areas covered 38.9 billion population, locates in the southwest of China. In Guizhou, 46.9 % of residents live in rural areas as of 2020; and residents have an average life expectancy of 74.2 years, a maternal mortality rate of 19.5 per 100,000 population and infant mortality rate of 7.5 ‰ in 2018. In 2018, there is a total of 1,369 PCFs and 755 PCFs in the rural and urban areas, respectively; and there is a total of 2.3 physicians and 3.0 nurse per 1000 population, respectively [33]. Per the national guideline for the capacity-building and quality improvement of PCFs issued in 2018 [34], only 133 PCFs in Guizhou met the criteria of basic standards of the national guideline as of 2019, which means only 133 PCFs can provide a wider service of basic medical services, preventive and public health services, such as internal medicine, surgical care, paediatrics services, gynaecology services, obstetrics services, general practice services, traditional Chinese Medicine (TCM) [35].

A multistage, stratified clustered sampling method was used to collect information about service scope of PCFs. We first randomly selected two cities from Guizhou, China per level of economic development based on the sampling method of one previous study [12]. Zunyi city has a higher level of economic development, while Tongren city is less developed. We then randomly selected two counties from each city (Sinan counties and Jiangkou counties from Tongren city, Meitan counties and Yuqing counties from Zunyi city) per the same principle. Yuqing county from Zunyi city has a higher level of economic development, while Meitan county from Zunyi city is less developed. Jiangkou county from Tongren city has a higher level of economic development, while Sinan county from Tongren city is less developed. (Appendix Table 1). Third, service scope of PCFs in 2017 was collected by a web-based survey with self-administrated questionnaires under the coordination of chief or deputy chief of each facility [16]. In this study, because some communities located between the urban and rural areas, PCFs located in these commnuties also served the rural residents. Therefore, a total of 57 rural PCFs and 7 urban PCFs were included. Fourth, per one previous study [6], claim data of 299,633 inpatient cases covered by 64 PCFs in 2017 was derived from the local information system of New Rural Cooperation Medical Scheme, which is generally purchased by residents living in the rural China.

### Outcome variable

Primary outcomes were (1) level of inpatient institutions (1 = PCFs, 2 = county-level hospitals, 3 = city-level hospitals, 4 = provincial hospitals), (2) length of stay, (3) per capita total health cost, (4) per capita out-of-pocket cost, (5) reimbursement ratio, (6) 30-day readmission [18–20]. The per capital total health cost is calculated from the supply side, which means how many cost occurred during an episode of inpatient sevices; and per capita out-of-pocket cost is how much did one patient spend during an episode of inpatient sevices. reimbursement ratio = 1-(per capita out-of-pocket cost/per capita total health cost)*100 %.

### Independent variable

Per our previous study, the independent variable of this study was facility-level service scope divided into preventive and public health services, and basic medical care services [17]. Preventive and public health services are consisted of (1) residents’ health records, (2) health education, (3) vaccination, (4) health management of children aged 0–6, (5) maternal health care, (6) health management of elderly people, (7) chronic disease management, (8) health management of patients with severe mental disorders, (9) health management of tuberculosis patients, (10) health management by TCM 11) reporting of and response to infectious disease and public health emergencies, and 12) health inspection and supervision. Basic medical care services are consisted of (1) internal medicine, (2) surgical care, (3) paediatrics services, (4) gynaecology services, (5) obstetrics services, (6) dental care, (7) referee services, (8) home care, (9) telemedicine services, (10) general practice services, 11) family practice services, 12) TCM, 13) rehabilitation services, 14) mental health services, 15) ED services, 16) hospice care, 17) basic anaesthesiology for minor procedures, 18) medical laboratory services, 19) medical imaging services, and 20) electrocardiography services [16]. The self-administrated questionnaire has previously been published in elsewhere [17]. The service scope score was calculated per cumulative service scope by PCFs, ranging from 1 to 32 [17].

### Control variables

In this study, covariates were age group, gender, poverty or not, having Critical Illness Insurance or not, referral, per capita total cost to represent the severity of the disease. We used the length of stay to represent the severity of the disease when outcome variables were cost-related indicators. Per capita total health cost was used as a covariate as illness severity with regression models when outcome variables were not cost-related indicators [18–20, 36].

### Statistical analysis

A total of 64 PCFs was categorized into five groups per facility-level service scope. Chi-squared tests and Fisher’s exact tests, independent t-tests were used to compare patient outcomes between PCFs within different groups; the Kruskal-Wallis tests followed by Dunn’s pairwise comparison were used to estimate the differences between different groups when the outcome variables are not normally distributed. Given the fact that limited higher-level sample size (a sample of 50 or less) could lead to biased estimates of the second-level standard errors for the two-level regression model [37], and some missing values in the facility-level factors (29 of 64 primary care facilities did not provide us any information about total number of staff, total financial revenue, direct government subsidies), we used the ordinary least squares regression models to examine the association between the service scope of PCFs and patient outcomes (Appendix 1). Given that cost data was skewed distributed, generalized linear models with a gamma distribution and log link function were used to estimate the marginal associations between the service scope of PCFs and patient outcomes. An ordinary logit model was conducted to estimate the association between service scope of PCFs and patients’ choice of inpatient institutions with different levels. Estimation models were shown in the Appendix (1) For the outcome variable of 30-day readmission, ordinal logistic regression model was used. Multicollinearity between various variables was assessed with the variance inflation factor (VIF > 10). In this study, VIFs of all regression models are both less than (2) All procedures were conducted with Stata 14.0. P < 0.05 was set to indicate statistical significance.

## Results

### Basic characteristic

As shown in Table 1, a total of 299,633 inpatient cases occurred in four counties in 2017; more than 20 % of inpatient cases are the elderly. Nearly 60 % of inpatient cases are female; 13.6 % of inpatient cases are under poverty status. A total 6.2 % of inpatient cases are covered by the Critical Illness Insurance. Differences on the age (χ^2^ = 770.6, P < 0.001), gender (χ^2^ = 130.0, P < 0.001), poverty status (χ^2^ = 725.1, P < 0.001), referral or not (χ^2^ = 542.3, P < 0.001), and Critical Illness Insurance (χ^2^ = 20.0, P < 0.001) are statistically significant.

**Table 1:** Basic characteristic of enrolled patients by facilities grouped by service scope, 2017

Variables	Service scope						χ^2^	P
	**Overall**	**Quantile 1**	**Quantile 2**	**Quantile 3**	**Quantile 4**	**Quantile 5**		
**Variables**	299,633 (100.0)	58,636 (19.6)	50,539 (16.9)	81,332 (27.1)	39,637 (13.2)	69,489 (23.2)		
**Age group**							770.6	< 0.001
< 18	56,322 (18.8)	10,616 (18.1)	8,681 (17.2)	15,425 (19.0)	6,616 (16.7)	14,984 (21.6)		
18–29	33,030 (11.0)	6,415 (10.9)	5,362 (10.6)	9,341 (11.5)	4,356 (11.0)	7,556 (10.9)		
30–44	49,123 (16.4)	10,099 (17.2)	8,425 (16.7)	13,255 (16.3)	6,381 (16.1)	10,963 (15.8)		
45–64	92,353 (30.8)	18,578 (31.7)	16,319 (32.3)	24,969 (30.7)	12,571 (31.7)	19,916 (28.7)		
> 64	68,805 (23.0)	12,928 (22.0)	11,752 (23.3)	18,342 (22.6)	9,713 (24.5)	16,070 (23.1)		
**Gender (%)**							130.0	< 0.001
Male	125,103 (41.8)	23,564 (40.2)	20,889 (41.3)	33,850 (41.6)	16,764 (42.3)	30,036 (43.2)		
Female	174,530 (58.3)	35,072 (59.8)	29,650 (58.7)	47,482 (58.4)	22,873 (57.7)	39,453 (56.8)		
**Poverty (%)**							725.1	< 0.001
Yes	40,696 (13.6)	9,528 (16.2)	5,427 (10.7)	10,705 (13.2)	5,332 (13.5)	9,704 (14.0)		
No	258,937 (86.4)	49,108 (83.8)	45,112 (89.3)	70,627 (86.8)	34,305 (86.5)	59,785 (86.0)		
**Referral (%)**							542.3	< 0.001
Yes	18,424 (6.2)	4,815 (8.2)	2,948 (5.8)	4,588 (5.6)	2,231 (5.6)	3,842 (5.5)		
No	281,209 (93.9)	53,821 (91.8)	47,591 (94.2)	76,744 (94.4)	37,406 (94.4)	65,647 (94.5)		
**Critical Illness Insurance (%)**							20.0	< 0.001
Yes	18,551 (6.2)	3,444 (5.9)	3,058 (6.1)	5,145 (6.3)	2,452 (6.2)	4,452 (6.4)		
No	281,082 (93.8)	55,192 (94.1)	47,481 (93.9)	76,187 (93.7)	37,185 (93.8)	65,037 (93.6)		

Note: Age group: 1 < 2, χ^2^ = 41.4, P < 0.001; 1 < 3, χ^2^ = 54.5, P < 0.001; 1 < 4, χ^2^ = 106.8, P < 0.001; 1 < 5, χ^2^ = 344.0, P < 0.001; 2 < 3, χ^2^ = 110.5, P < 0.001; 2 < 4, χ^2^ = 27.4, P < 0.001; 2 < 5, χ^2^ = 430.0, P < 0.001; 3 < 4, χ^2^ = 134.8, P < 0.001; 3 < 5, χ^2^ = 202.0, P < 0.001; 4 < 5, χ^2^ = 404.0, P < 0.001. Gender: 1 < 2, χ^2^ = 14.8, P < 0.001; 1 < 3, χ^2^ = 14.8, P < 0.001; 1 < 4, χ^2^ = 43.4, P < 0.001; 1 < 5, χ^2^ = 120.6, P < 0.001; 2 < 4, χ^2^ = 8.4, P = 0.004; 2 < 5, χ^2^ = 42.9, P < 0.001; 3 < 5, χ^2^ = 5.0, P = 0.026; 4 < 5, χ^2^ = 8.9, P = 0.003. Poverty: 1 < 2, χ^2^ = 697.4, P < 0.001; 1 < 3, χ^2^ = 262.6, P < 0.001; 1 < 4, χ^2^ = 144.2, P < 0.001; 1 < 5, χ^2^ = 130.1, P < 0.001; 2 < 3, χ^2^ = 170.6, P < 0.001; 2 < 4,χ^2^ = 155.7, P < 0.001; 2 < 5,χ^2^ = 276.5, P < 0.001; 3 < 4, χ^2^ = 134.8, P < 0.001; 3 < 5, χ^2^ = 20.6, P < 0.00; 4 < 5, χ^2^ = 5.6, P = 0.018. Referral: 1 < 2, χ^2^ = 232.5, P < 0.001; 1 < 3, χ^2^ = 359.3, P < 0.001; 1 < 4, χ^2^ = 363.3, P < 0.001; 2 < 5, χ^2^ = 363.3, P = 0.024; Critical Illness Insurance: 1 < 3, χ^2^ = 12.1, P < 0.001; 1 < 4, χ^2^ = 4.1, P < 0.043; 1 < 5, χ^2^ = 15.6, P < 0.001; 2 < 3, χ^2^ = 4.0, P < 0.044; 2 < 5, χ^2^ = 6.3, P < 0.012

As shown in Table 2, A total of 12.0 % of inpatient cases occurred in the city-level or provincial hospitals; 4.9 % of inpatient patients were readmitted within 30 days, the median of length of stay was 6 days, per capital total cost was 1,873.1 Chinese Yuan, per capita out-of-pocket cost was 663.8 Chinese Yuan within a reimbursement ratio of 63.6 %. Differences on the level of inpatient institution (χ^2^ = 5600.0, P < 0.001), readmission in 30 days (χ^2^ = 31.2, P < 0.001), length of stay (U = 535.9, P < 0.001), per capita total cost (U = 1,202.3, P < 0.001), per capita out-of-pocket cost (U = 1,756.8, P < 0.001), reimbursement ratio (U = 1,720.4, P < 0.001) between different groups of service scope are statistically significant. Detailed service scope of sample primary care facilities and comparsion by counties was shown in the Appendix Table 2 and Appendix Table 3. Basic characteristics and patient outcomes of enrolled patients by counties are shown in Appendix Table 4 and Appendix Table 5.

**Table 2:** Patient outcomes of enrolled patients by facilities grouped by service scope, 2017

Variables	Service scope					U/χ^2^	P
	**Quantile 1**	**Quantile 2**	**Quantile 3**	**Quantile 4**	**Quantile 5**		
**Level of inpatient institution(%)**							
PCF-level	17,546 (29.9)	14,757 (29.2)	24,244 (29.8)	12,757 (32.2)	29,884 (43.0)	5600.0	< 0.001
County-level	34,083 (58.1)	31,065 (61.5)	48,864 (60.1)	22,919 (57.8)	31,335 (45.1)		
City-level	4,141 (7.1)	1,937 (3.8)	3,763 (4.6)	1,775 (4.5)	4,410 (6.3)		
Provincial	2,866 (4.9)	2,780 (5.5)	4,461 (5.5)	2,186 (5.5)	3,860 (5.6)		
**Readmission in 30 days**							
Yes	2893 (4.9)	2267 (4.5)	3560 (4.4)	1763 (4.4)	3039 (4.4)	31.2	< 0.001
No	58,636 (95.1)	50,539 (95.5)	81,332 (95.6)	39,637 (95.6)	69,489 (95.6)		
**Length of stay (Median, [p25, p75])**	6 (4, 8)	5 (3, 8)	6 (4, 8)	5 (4, 8)	6 (4, 8)	535.9	< 0.001
**Length of stay (Mean ± SD)**	7.8 ± 13.7	7.3 ± 14.0	7.3 ± 11.7	7.4 ± 12.2	7.8 ± 12.9		
**Per capita total cost** **(In Chinese Yuan)**	1,873.1 (1066.0, 3,754.1)	1,652.5 (892.5, 3,344.7)	1617.5 (857.0, 3,388.8)	1,610.4 (869.5, 3,311.8)	1,684.5 (920.2, 3,460.5)	1,202.3	< 0.001
**Per capita out-of-pocket cost** **(In Chinese Yuan)**	663.8 (317.4, 1,330.8)	559.7 (231.0, 1,177.7)	540.7 (217.3, 1,169.9)	534.7 (221.4, 1,153)	545.9 (227, 1,144.5)	1,756.8	< 0.001
**Reimbursement ratio (%)**	63.6 ± 17.9	66.1 ± 39.0	66.8 ± 64.8	66.6 ± 21.2	66.6 ± 21.2	1,720.4	< 0.001

Note: Level of inpatient institution (%): 1 < 2, χ^2^ = 583.8, P < 0.001; 1 < 3, χ^2^ = 403.2, P < 0.001; 1 < 4, χ^2^ = 320.0, P < 0.001; 1 < 5, χ^2^ = 2600.0, P < 0.001; 2 < 3, χ^2^ = 59.3, P < 0.001; 2 < 4, χ^2^ = 136.7, P < 0.001; 2 < 5, χ^2^ = 3400.0, P < 0.001; 3 < 4, χ^2^ = 73.4, P < 0.001; 3 < 5, χ^2^ = 3600.0, P < 0.00; 4 < 5, χ^2^ = 1700.0, P < 0.001. Readmission in 30 days: 1 < 2, χ^2^ = 11.0, P = 0.001; 1 < 3, χ^2^ = 21.9, P < 0.001; 1 < 4, χ^2^ = 11.3, P = 0.001; 1 < 5, χ^2^ = 20.6, P < 0.001. Length of stay (Median, [p25, p75]): 1 > 2: U = 7.6, P < 0.001; 1 > 4: U = 2.7, P = 0.004; 1 < 5, U = -14.1, P < 0.001; 2 < 3, U = -6.9, P < 0.001; 2 < 4, U = − 4.3, P < 0.001; 2 < 5, U = -21.3, P < 0.001; 3 > 4, U = 1.72, P = 0.043; 3 < 5, U = 16.6, P = 0.001; 4 < 5, U=-15.3, P < 0.001. Per capita total cost (In Chinese Yuan): 1 > 2: U = 25.2, P < 0.001; 1 > 3: U = 31.1 P < 0.001; 1 > 4: U = 26.8, P < 0.001; 1 > 5: U = 21.1, P < 0.001; 2 > 3: U = 2.76, P = 0.003; 2 > 4: U = 3.2, P < 0.001; 2 < 5: U = -5.84, P < 0.001; 3 < 5: U = -9.63, P < 0.001; 4 < 5: U = -8.8, P < 0.001. Per capita out-of-pocket cost (In Chinese Yuan): 1 > 2: U = 28.6, P < 0.001; 1 > 3: U = 36.9, P < 0.001; 1 > 4: U = 31.4, P < 0.001; 1 > 5: U = 33.4, P < 0.001; 2 > 3: U = 4.6, P < 0.001; 2 > 4: U = 4.6, P < 0.001; 2 > 5: U = 33.4, P = 0.009; 3 < 5: U = -2.4, P = 0.009; 4 < 5: U = -2.7, P = 0.003. Reimbursement ratio (%): 1 < 2: U = -22.3, P < 0.001; 1 < 3: U = -33.9, P < 0.001; 1 < 4: U = -27.5, P < 0.001; 1 < 5: U = -37.9, P < 0.001; 2 < 3, U = -8.5, P < 0.001; 2 < 4, U = -6.5, P < 0.001; 2 < 5, U=-5.6, P < 0.001; 3 < 5, U = -5.6, P < 0.001; 4 < 5, U = -5.3, P < 0.001

### Association between service scope of primary care facilities and patient outcomes

As shown in Table 3, after controlling demographic and clinical covariates, patients living in the communities with PCFs of greatest service scope were less likely to be admitted into the county-level hospitals (Quantile 5 vs. Quantile 1: Marginal difference[95 % CI]: -6.19 % [-6.49 %, -5.89 %]), city-level hospitals (Quantile 5 vs. Quantile 1: Marginal difference[95 % CI]: -1.88 % [-1.97 %, -1.78 %]) and provincial hospital (Quantile 5 vs. Quantile 1: Marginal difference[95 % CI]: -2.11 % [-2.22 %, -2.00 %]) than their counterparts living in the communities with PCFs of least service scope. As shown in Table 4, after controlling other covariates, patients living in the communities with PCFs of greatest service scope were less likely to be re-admitted within 30 days (Quantile 5 vs. Quantile 1: Marginal difference [95 % CI]: -0.45 % [-0.68 %, -0.22 %]) with an equal length of stay (Quantile 5 vs. Quantile 1: Marginal difference [95 % CI]: -0.02 [-0.16, 0.11]) than their counterparts living in the communities with facilities of least service scope. Meanwhile, patients living in the communities with facilities of greatest service scope spent less both in the total cost (Quantile 5 vs. Quantile 1: Marginal difference [95 % CI]: -201.8 [-257.9, -145.8]) and out-of-pocket cost (Quantile 5 vs. Quantile 1: Marginal difference [95 % CI]: -210.2 [-237.3, -183.2]), and had a greater reimbursement ratio (Quantile 5 vs. Quantile 1: Marginal difference [95 % CI]: 2.3 % [1.9 %, 2.8 %]) than their counterparts living in the communities with PCFs of least service scope.

**Table 3:** Marginal differences of facility-level service scope on patients’ choice on the level of inpatient institution, 2017

Variables		Level of inpatient institution
		**Marginal differences (%) (95 % CI)**
**Quantile 2(vs. Quantile 1)**	PCF-level	0.78(0.29, 1.27)
**Quantile 3(vs. Quantile 1)**		0.86(0.42, 1.29)
**Quantile 4(vs. Quantile 1)**		2.59(2.05, 3.12)
**Quantile 5(vs. Quantile 1)**		**10.18(9.70, 10.67)**
**Quantile 2(vs. Quantile 1)**	County-level	-0.40(-0.64, -0.15)
**Quantile 3(vs. Quantile 1)**		-0.44(-0.66, -0.21)
**Quantile 4(vs. Quantile 1)**		-1.38(-1.66, -1.09)
**Quantile 5(vs. Quantile 1)**		**-6.19(-6.49, -5.89)**
**Quantile 2(vs. Quantile 1)**	City-level	-0.18(-0.29, -0.06)
**Quantile 3(vs. Quantile 1)**		-0.19(-0.29, -0.09)
**Quantile 4(vs. Quantile 1)**		-0.56(-0.67, -0.44)
**Quantile 5(vs. Quantile 1)**		**-1.88(-1.97, -1.78)**
**Quantile 2(vs. Quantile 1)**	Provincial	-0.21(-0.34, -0.08)
**Quantile 3(vs. Quantile 1)**		-0.23(-0.34, -0.11)
**Quantile 4(vs. Quantile 1)**		-0.65(-0.78, -0.52)
**Quantile 5(vs. Quantile 1)**		**-2.11(-2.22, -2.00)**

Note: age group, gender, poverty, referral, Critical Illness Insurance and total cost were set as covariates

**Table 4:** Marginal differences of facility-level service scope on patients’ 30-days readmission, cost, length of stay and reimbursement ratio, 2017

Variables	30-days readmission*	Length of stay*	Per capita total cost**	Per capita total out-of-pocket cost**	Reimbursement ratio **
	**Marginal differences (%) (95 % CI)**				
**Quantile 2(vs. Quantile 1)**	-0.24(-0.49, 0.01)	-0.08(-0.22, 0.07)	-279.2(-339.1, -219.3)	-137.8(-167.3, -108.3)	1.8(1.3, 2.2)
**Quantile 3(vs. Quantile 1)**	-0.42(-0.64, -0.20)	-0.44(-0.57, -0.31)	-295.7(-349.4, -241.9)	-188.7(-215.0, -162.3)	2.5(2.1, 2.9)
**Quantile 4(vs. Quantile 1)**	-0.39(-0.65, -0.12)	-0.46(-0.61, -0.31)	-322.7(-386.1, -259.3)	-190.6(-221.6,-159.7)	2.3(1.8, 2.8)
**Quantile 5(vs. Quantile 1)**	-0.45(-0.68, -0.22)	-0.02(-0.16, 0.11)	-201.8(-257.9, -145.8)	-210.2(-237.3,-183.2)	2.3(1.9, 2.8)

Note: *age group, gender, poverty, referral, Critical Illness Insurance and total cost were set as covariates; ** age group, gender, poverty, referral, Critical Illness Insurance and length of stay were set as covariates

## Discussion

To the best of our knowledge, this study is the first study to examine association between service scope of PCFs and patient outcomes in China. Understanding marginal changes in patient outcomes associated with service scope of PCFs might inform policymakers on how to strengthen the current primary care system and develop tailored and feasible interventions more effectively. PCFs’ service scope in rural Guizhou, China varied a lot, which is consistent with the national level reported by one previous study [17]. Significant disparities in inpatient services utilization, quality of care and cost also indcates potential benefits to strengthening the current primary care system.

First, patients living in the communities with PCFs with greater service scope were more likely to be admitted into the PCFs. It may be related to improved accessibility to care provided by PCFs for patients with common illness or continuity of care discharged from high-level hospitals. These results might also be caused by a greater reimbursement ratio for inpatient services provided by PCFs than high-level hospitals, which are more attractive to low-income residents [11, 13]. These results are similar to findings of previous studies in the US that expanded service scope of nurse practitioners could reduce unnecessary utilization of hospitalization [5], and comprehensive care by family practitioners is associated with reduced utilization of services and decreased cost [6].

Second, a total of 4.9 % of patients were re-admitted in 30 days. This result is higher than the estimates (3.3 %) of one previous study in a county from rural Guizhou [38]. Our results indicated that the greater service scope of PCFs is associated with lower 30-day readmission rates. It would mean that improved service scope of PCFs is associated with increased quality of care from the perspective of readmission. Meanwhile, smaller service of scope by PCFs might lead to a greater 30-day readmission rate due to limited diagnosis capacity. In addition, association between the servicc scope of PCFs and patients’ length of stay is nonlinear. It might be related to the fact that PCFs with greatest service scope could served more patients with more severe diseases that need a greater length of stay. While PCFs with moderate service scope would also attract some patients to use inpatient services in the PCFs, instead of going to hospitals directly. It may also be related to the fact that physician in secondary and tertiary hospitals were given incentives to achieve maximum profits, which might cause a greater length of stay in some cases [39]. One previous study pointed out that the educational intervention programs could improve the quality of care for child upper respiratory tract infections in resource-poor settings [40]. Expansion of telehealth for stroke services could also improve the quality of care provided in super rural areas [41]. Meanwhile, nurse practitioners could provide health care services with comparable quality of care with an even lower cost when nurse practitioners practiced independently [9]. Even the effect of removing restrictive scope-of-practice laws on the primary care workforce’s capacity was modestly in the short run [42], regulation restricting scope-of-practice for nurse practitioners does not improve quality of care [42]. These experiences remind us in the rural and remote areas, extending the scope of PCFs could be started from education and training of primary care providers, innovative healthcare delivery initiatives, and eliminating scope-of-practice policies and laws, thus mitigating the shortage of primary care physicians.

Third, we also found that PCFs’ greater service scope were also associated with reduced per capita total cost and per capita out-of-pocket cost and increased reimbursement ratio. It might be related to the fact that the hospitals’ fee-for-service payment system would incentive unnecessary testing and treatment [31]. This result is consistent with findings of one previous study that healthcare provided by the retail clinic was associated with a lower cost of per episode [9]. These results might also lead to greater satisfaction among residents [43]. Meanwhile, the autonomy of primary care practitioners is also related to their satisfaction and intention to stay in their jobs [44]. These findings indicate that it is urgent to change patient’s preference for inpatient services both from the availability and affordability of services provided by PCFs in rural China, thus inducing utilization of services provided by PCFs [45]. However, the transition to innovative care initiatives, such as patient-centered medical homes, is challenging for small facilities, which raises concerns about the appropriateness of service scope expected from the primary care providers and calls for external supports, such as practice design, payment reform and health information technologies [46, 47].

## Limitation

This study has several limitations. First, self-reported service scope may be subject to social desirability bias. To control the bias of self-reported survey, officers from the local health departments were compensated to help check the accuracy of reported data by PCFs; and the research team also double checked the data and then entered these data into the final dataset. Second, Guizhou is a less developed province in China, which indicates that the current findings might be limited in those areas with resource-limited settings. Third, we could not make the causal inference based on the cross-sectional study, and future studies should conduct intervention trials to determine whether expanding service scope of PCFs could increase utilization of primary care services, improve quality of care and achieve the goal of cost-savings or not. In addition, areas for future research include cost-effectiveness analysis of strengthening the service scope of PCFs with long-term health outcomes.

## Conclusions

This study revealed the association between the service scope of PCFs and patient outcomes in rural Guizhou, China. These findings demonstrate the potential to increase utilization of primary care services, quality of care and cost-savings by extending the service scope of PCFs, which would be useful for policymakers, institution manager, and administrator of medical security to attract more patients to use services provided by PCFs, especiallty those in the rural areas with limited healthcare resources. To effectively meet primary care needs in rural China, policymakers and healthcare providers should appropriately enact more tailored support for rural physician practices.

## Supplementary information

**Additional file 1**

## Data Availability

All the research data is available from the corresponding author upon reasonable request.

## Abbreviations

PCF: Primary care facilities
TCM: Tranditional Chinese Medicine
VIF: Variation inflation factor
US: United States
