# Supplementary material for: Association between service scope of primary care facilities and patient outcomes: a retrospective study in rural Guizhou, China
Source: BMC Health Serv Res. 2021 Aug 28;21:885. doi: 10.1186/s12913-021-06877-4 (PMC8400844; doi:10.1186/s12913-021-06877-4)
Supplement: Supplementary file 1 — Additional file 1 [file 12913_2021_6877_MOESM1_ESM.docx]

# **Appendix Table 1. Socioeconomic and demographic indicators of four sampled counties in rural Guizhou, 2020**

| **Indicators** | **Sinan,**  **Tongren** | **Jiangkou,**  **Tongren** | **Meitan,**  **Zunyi** | **Yuqing,**  **Zunyi** |
| --- | --- | --- | --- | --- |
| **Number of residents** | 457,745 | 184,764 | 372,865 | 223,952 |
| Male | 229,795 | 95,489 | 185,494 | 111,792 |
| Female | 227,950 | 89,275 | 187,371 | 112,160 |
| **Age structure of the residents** |  |  |  |  |
| 0-14 | 22.8 | 23.4 | 21.0 | 22.4 |
| 15-59 | 56.9 | 58.1 | 60.0 | 57.3 |
| ≥ 60 | 20.4 | 18.5 | 19.3 | 20.4 |
| ≥ 65 | 16.7 | 14.0 | 15.6 | 16.6 |
| **Per capita GDP (in 10,000 Chinese Yuan)** | 2.54 | 3.22 | 2.74 | 3.50 |
| **Number of township-level or street-level communities** | 29 | 17 | 15 | 10 |
| **Number of villages** | 489 | 104 | 119 | 56 |
| **Number of public hospitals** | 2 | 3 | 2 | 13 |
| **Number of private hospitals** | 12 | 6 | 9 | 3 |
| **Primary care facilities sampled** | 29 | 10 | 15 | 10 |

Note: county-level basic health indicators of residents are not calculated. Therefore, we collected the city-level basic health indicators as follows. Resident in Zunyi city has an average life expectancy of 72.0 years and resident in Tongren city has an aveage life expectancy of 71.2 years in 2010. Neonatal mortality, infant mortality rate, under five mortality, maternal mortality is 2.18‰, 4.46‰, 6.36‰ and 14.35 per 100,0000 population in Zunyi city in 2019. Neonatal mortality, infant mortality rate, under five mortality, maternal mortality is 2.18‰, 5.7‰, 7.78‰ and 21.99 per 100,0000 population in Tongren city in 2018.

# **Appendix 1. Estimation models**

**1) Level of inpatient institutions as outcome variable:**

Let *i* = 1, 2, …, N indexes the patients. Levelofinpatientinstitution captures level of inpatient institutions (1= PCF-level, 2=county-level, 3 = city-level, 4 = provincial), servicescope is an ordinary variable that indicates which group that patients living in communities with PCFs of different service scope (1=quantile 1, 2= quantile 2, 3= quantile 3, 4= quantile 4, 5= quantile 5). $\beta_{1}$ is coefficients to be estimated. Z is the control variables, in this model, it includes age group, gender, poverty or not, referral, having Critical Illness Insurance or not and per capita total health cost.

**2) Readmission in 30 days as outcome variable:**

Let *i* = 1, 2, …, N indexes the patients. Readmission30days captures whether the patient was readmitted within 30 days after previous discharge from hospitals (0= no, 1=yes), servicescope is an ordinary variable that indicates which group that patients living in communities with PCFs of different service scope (1=quantile 1, 2= quantile 2, 3= quantile 3, 4= quantile 4, 5= quantile 5). $\beta_{1}$ is coefficients to be estimated. Z is the control variables, in this model, it includes age group, gender, poverty or not, referral, having Critical Illness Insurance or not and per capita total health cost.

**3）Length of stay as outcome variable:**

Let i = 1, 2, …, N indexes the patients. LOS captures length of stay that occurred during one inpatient services. Servicescope is an ordinary variable that indicates which group that patients living in communities with PCFs of different service scope (1=quantile 1, 2= quantile 2, 3= quantile 3, 4= quantile 4, 5= quantile 5). $\beta_{1}$ is coefficients to be estimated. Z is the control variables, in this model, it includes age group, gender, poverty or not, referral, having Critical Illness Insurance or not and per capita total health cost.

**4）Per capita total cost, Per capita out-of-pocket cost, Reimbursement ratio as outcome variables:**

Let i = 1, 2, …, N indexes the patients. Totalcost captures Per capita total cost that occurred during one inpatient services. Totaloopcost captures Per capita total out-of-pocket cost that occurred during one inpatient services. Reimbursratio captures Reimbursement ratio that occurred during one inpatient services. Servicescope is an ordinary variable that indicates which group that patients living in communities with PCFs of different service scope (1= quantile 1, 2= quantile 2, 3= quantile 3, 4= quantile 4, 5= quantile 5). $\beta_{1}$ is coefficients to be estimated. Z is the control variables, in this model, it includes age group, gender, poverty or not, referral, having Critical Illness Insurance or not and length of stay.

# **Appendix Table 2. Service scope of sampled primary care facilities, 2017**

| **Service scope score** | **Frequencies** | **%** | **Cumulative**  **%** | **Meitan** | **Yuqing** | **Jiangkou** | **Sinan** |
| --- | --- | --- | --- | --- | --- | --- | --- |
| 13 | 1 | 1.6 | 1.6 | 0 | 0 | 1 | 0 |
| 15 | 1 | 1.6 | 3.1 | 0 | 1 | 0 | 0 |
| 16 | 6 | 9.4 | 12.5 | 1 | 0 | 2 | 3 |
| 17 | 7 | 10.9 | 23.4 | 0 | 0 | 1 | 6 |
| 18 | 3 | 4.7 | 28.1 | 2 | 0 | 0 | 1 |
| 19 | 7 | 10.9 | 39.1 | 1 | 1 | 0 | 5 |
| **20** | **9** | **14.1** | **53.1** | 4 | 0 | 1 | 4 |
| 21 | 9 | 14.1 | 67.2 | 1 | 3 | 2 | 3 |
| 22 | 9 | 14.1 | 81.3 | 4 | 1 | 0 | 4 |
| 23 | 6 | 9.4 | 90.6 | 1 | 0 | 3 | 2 |
| 25 | 1 | 1.6 | 92.2 | 1 | 0 | 0 | 0 |
| 26 | 4 | 6.3 | 98.4 | 0 | 3 | 0 | 1 |
| 27 | 1 | 1.6 | 100.0 | 0 | 1 | 0 | 0 |

Note: Wilcoxon rank-sum test was used to compare the differences of services scope provided by PCFs by counties; results indicated the differences is not statistically significant (χ^2^ = 6.23, P = 0.101).

# **Appendix Table 3. Primary care facilities with service items by counties, 2017**

| **Service items** | **Overall**  **(N=64)** | **Sinan (N=29)** | **Jiangkou**  **(N=10)** | **Meitan**  **(N=15)** | **Yuqing**  **(N=10)** |
| --- | --- | --- | --- | --- | --- |
|  | **N (%)** | **N (%)** | **N (%)** | **N (%)** | **N (%)** |
| residents’ health records | 64 (100.0) | 29 (100.0) | 10 (100.0) | 15 (100.0) | 10 (100.0) |
| health education | 64 (100.0) | 29 (100.0) | 10 (100.0) | 15 (100.0) | 10 (100.0) |
| vaccination | 64 (100.0) | 29 (100.0) | 10 (100.0) | 15 (100.0) | 10 (100.0) |
| health management of children aged 0–6 | 62 (96.9) | 28 (96.6) | 9 (90.0) | 15 (90.0) | 10 (100.0) |
| maternal health care | 63 (98.4) | 29 (100.0) | 9 (90.0) | 15 (90.0) | 10 (100.0) |
| health management of elderly people | 64 (100.0) | 29 (100.0) | 10 (100.0) | 15 (100.0) | 10 (100.0) |
| chronic disease management | 64 (100.0) | 29 (100.0) | 10 (100.0) | 15 (100.0) | 10 (100.0) |
| health management of patients  with severe mental disorders | 39 (60.9) | 19 (65.5) | 6 (60.0) | 7 (60.0) | 7 (70.0) |
| health management of tuberculosis patients | 56 (87.5) | 23 (79.3) | 9 (90.0) | 14 (90.0) | 10 (100.0) |
| health management by TCM | 63 (98.4) | 28 (96.6) | 10 (100.0) | 15 (100.0) | 10 (100.0) |
| reporting of and response to infectious disease  and public health emergencies | 64 (100.0) | 29 (100.0) | 10 (100.0) | 15 (100.0) | 10 (100.0) |
| health inspection and supervision | 60 (93.8) | 28 (96.6) | 8 (80.0) | 15 (80.0) | 9 (90.0) |
| internal medicine | 64 (100.0) | 29 (100.0) | 10 (100.0) | 15 (100.0) | 10 (100.0) |
| surgical care | 12 (18.8) | 5 (17.2) | 1 (10.0) | 1 (10.0) | 5 (50.0) |
| paediatrics services | 41 (64.1) | 21 (72.4) | 6 (60.0) | 7 (60.0) | 7 (70.0) |
| gynaecology services | 40 (62.5) | 19 (65.5) | 3 (30.0) | 12 (30.0) | 6 (60.0) |
| obstetrics services | 13 (20.3) | 7 (24.1) | 0 (0) | 2 (0) | 4 (40.0) |
| dental care | 12 (18.8) | 4 (13.8) | 1 (10.0) | 3 (10.0) | 4 (40.0) |
| referee services | 60 (93.8) | 28 (96.6) | 8 (80.0) | 15 (80.0) | 9 (90.0) |
| home care | 5 (7.8) | 1 (3.4) | 2 (20.0) | 1 (20.0) | 1 (10.0) |
| telemedicine services | 39 (60.9) | 13 (44.8) | 4 (40.0) | 13 (40) | 9 (90.0) |
| general practice services | 62 (96.9) | 29 (100.0) | 9 (90.0) | 15 (90.0) | 9 (90.0) |
| family practice services | 19 (29.7) | 3 (10.3) | 5 (50.0) | 7 (50.0) | 4 (40.0) |
| TCM | 46 (71.9) | 16 (55.2) | 8 (80.0) | 13 (80.0) | 9 (90.0) |
| rehabilitation services | 0 (0) | 0 (0) | 0 (0) | 0 (0) | 0 (0) |
| mental health services | 0 (0) | 0 (0) | 0 (0) | 0 (0) | 0 (0) |
| ED services | 47 (73.4) | 21 (72.4) | 6 (60.0) | 11 (60.0) | 9 (90.0) |
| hospice care | 2 (3.1) | 0 (0) | 1 (10.0) | 1 (10.0) | 0 (0) |
| basic anaesthesiology for minor procedures | 14 (21.9) | 6 (20.7) | 2 (20.0) | 1 (20.0) | 5 (50.0) |
| medical laboratory services | 10 (15.6) | 4 (13.8) | 2 (20.0) | 2 (20.0) | 2 (20.0) |
| medical imaging services | 14 (21.9) | 2 (6.9) | 4 (40.0) | 3 (40.0) | 5 (50.0) |
| electrocardiography services | 64 (100.0) | 29 (100.0) | 10 (100.0) | 15 (100.0) | 10 (100.0) |

# **Appendix Table 4. Basic characteristic of enrolled patients by county, 2017**

| **Variables** | **Overall (%)** | **Meitan** | **Yuqing** | **Jiangkou** | **Sinan** |
| --- | --- | --- | --- | --- | --- |
| **Overall** | 299,633 (100.0) | 75,202 (25.1) | 54,939 (18.3) | 40,903 (13.7) | 128,589 (42.9) |
| **Age group** |  |  |  |  |  |
| < 18 | 56,322 (18.8) | 15,065 (20) | 12,154 (22.1) | 8,894 (21.7) | 20,209 (15.7) |
| 18-29 | 33,030 (11.0) | 9,814 (13.1) | 5,363 (9.8) | 5,273 (12.9) | 12,580 (9.8) |
| 30-44 | 49,123 (16.4) | 10,612 (14.1) | 8,868 (16.1) | 6,946 (17.0) | 22,697 (17.7) |
| 45-64 | 92,353 (30.8) | 21,169 (28.1) | 15,976 (29.1) | 11,092 (27.1) | 44,116 (34.3) |
| > 64 | 68,805 (23.0) | 18,542 (24.7) | 12,578 (22.9) | 8,698 (21.3) | 28,987 (22.5) |
| **Gender (%)** |  |  |  |  |  |
| Male | 125,103 (41.8) | 33,868 (45.0) | 23,785 (43.3) | 17,660 (43.2) | 49,790 (38.7) |
| Female | 174,530 (58.3) | 41,334 (55.0) | 31,154 (56.7) | 23,243 (56.8) | 78,799 (61.3) |
| **Group by service scope (%)** |  |  |  |  |  |
| Quantile 1 (< 18) | 58,636 (19.6) | 3,548 (4.7) | 4,090 (7.4) | 16,429 (40.2) | 34,569 (26.9) |
| Quantile 2 (18-20) | 50,539 (16.9) | 15,463 (20.6) | 8,898 (16.2) | 0 (0) | 26,178 (20.4) |
| Quantile 3 (20-21) | 81,332 (27.1) | 30,296 (40.3) | 11,258 (20.5) | 9,756 (23.9) | 30,022 (23.3) |
| Quantile 4 (22) | 39,637 (13.2) | 13,750 (18.3) | 6,858 (12.5) | 0 (0) | 19,029 (14.8) |
| Quantile 5 (> 22) | 69,489 (23.2) | 12,145 (16.1) | 23,835 (43.4) | 14,718 (36.0) | 18,791 (14.6) |
| **Poverty (%)** |  |  |  |  |  |
| Yes | 40,696 (13.6) | 6,159 (8.2) | 4,791 (8.7) | 10,421 (25.5) | 19,325 (15.0) |
| No | 258,937 (86.4) | 69,043 (91.8) | 50,148 (91.3) | 30,482 (74.5) | 109,264 (85.0) |
| **Referral (%)** |  |  |  |  |  |
| Yes | 18,424 (6.2) | 1,534 (2.0) | 1,046 (1.9) | 3,624 (8.9) | 12,220 (9.5) |
| No | 281,209 (93.9) | 73,668 (98) | 53,893 (98.1) | 37,279 (91.1) | 116,369 (90.5) |
| **Critical Illness Insurance (%)** |  |  |  |  |  |
| Yes | 18,551 (6.2) | 5,144 (6.8) | 3,970 (7.2) | 2,657 (6.5) | 6,780 (5.3) |
| No | 281,082 (93.8) | 70,058 (93.2) | 50,969 (92.8) | 38,246 (93.5) | 121,809 (94.7) |

# **Appendix Table 5. Patient outcomes of enrolled patients by county, 2017**

| **Variables** | **Overall (%)** | **Meitan** | **Yuqing** | **Jiangkou** | **Sinan** |
| --- | --- | --- | --- | --- | --- |
| **Overall** | 299,633 (100.0) | 75,202 (25.1) | 54,939 (18.3) | 40,903 (13.7) | 128,589 (42.9) |
| **Level of inpatient institutions（%）** |  |  |  |  |  |
| PCF-level | 99,188 (33.1) | 17,915 (23.8) | 25,319 (46.1) | 6,490 (15.9) | 49,464 (38.5) |
| County-level | 168,266 (56.2) | 49,647 (66.0) | 23,464 (42.7) | 26,303 (64.3) | 68,852 (53.5) |
| City-level | 16,026 (5.4) | 2,842 (3.8) | 1,675 (3.0) | 6,190 (15.1) | 5,319 (4.1) |
| Provincial | 16,153 (5.4) | 4,798 (6.4) | 4,481 (8.2) | 1,920 (4.7) | 4,954 (3.9) |
| **Readmission in 30 days** |  |  |  |  |  |
| Yes | 13,522 (4.5) | 13,522 (4.5) | 2,612 (3.5) | 2,187 (4.0) | 2,615 (5.3) |
| No | 286,111(95.5) | 286,111(95.5) | 75,202 (96.5) | 52,752 (96.0) | 38,738 (94.7) |
| **Length of stay (Median, [p25, p75])** | 6 (4,8) | 6 (4,8) | 6 (4,8) | 6 (5,9) | 5 (3,8) |
| **Length of stay (Mean ± SD)** | 7.6 ± 12.9 | 7.6 ± 12.0 | 7.6 ± 12.4 | 8.4 ± 10.5 | 7.3 ± 14.1 |
| **Per capita total cost (In Chinese Yuan)** | 1688.9 (914.8, 3466.6) | 1189.3 (519.9, 2630.3) | 1564.0 (865.7, 3225.0) | 2145.1 (1154.7, 4215.5) | 1876.3 (1112.9, 3667.2) |
| **Per capita out-of-pocket cost (In Chinese Yuan)** | 571 (238.5, 1196.0) | 373.2 (72.0, 870.1) | 395.9 (183.5, 933.5) | 696.8 (392.5, 1380.5) | 712.9 (348.4, 1388.1) |
| **Reimbursement ratio (%)** | 66.0 ± 40.3 | 70.8 ± 25.0 | 70.5 ± 23.2 | 64.2 ± 19.0 | 61.8 ± 55.0 |
